# Events associated with susceptibility to invasive *Salmonella enterica* serovar Typhi in BALB/c mice previously infected with *Plasmodium berghei* ANKA

**DOI:** 10.1038/s41598-021-82330-0

**Published:** 2021-02-01

**Authors:** Yasmin Cabral Moreira, Maele Jordão, Oscar Tadeu Ferreira da Costa, Elizangela Farias, Alysson Guimaraes Costa, Viviane de Farias, Dorval Antonio Mafra Coimbra, Tatiana Bacry Cardoza, Yury Oliveira Chaves, Patricia Puccinelli Orlandi, Fabio Trindade Maranhão Costa, Paulo Afonso Nogueira

**Affiliations:** 1 Fundação Oswaldo Cruz-Fiocruz, Instituto Leônidas e Maria Deane (ILMD- Fiocruz Amazônia), Manaus, AM Brazil; 2 Programa de Pós-Graduação em Biologia da Interação Patógeno Hospedeiro, ILMD- Fiocruz Amazônia, Manaus, AM Brazil; 3 Instituto de Ciências Biológicas, Universidade Federal do Amazonas, Manaus, AM Brasil; 4 Diretoria de Ensino e Pesquisa, Fundação Hospitalar de Hematologia e Hemoterapia do Amazonas, Manaus, AM Brazil; 5 Fundação de Medicina Tropical Dr Heitor Vieira Dourado, Instituto de Pesquisa Clínica Carlos Borborema, Manaus, AM Brazil; 6 Programa de Pós-Graduação em Imunologia Básica e Aplicada, Universidade Federal do Amazonas, Manaus, AM Brasil; 7 Fundação Oswaldo Cruz-Fiocruz, Programa de Pós-graduação Stricto sensu em Biologia Parasitária do Instituto Oswaldo Cruz, Rio de Janeiro, RJ Brazil; 8 Laboratório de Doenças Tropicais, Instituto de Biologia, Universidade Estadual de Campinas, Campinas, SP Brazil

**Keywords:** Immunology, Microbiology

## Abstract

Numerous mechanisms have been proposed to explain why patients with malaria are more susceptible to bloodstream invasions by *Salmonella* spp., however there are still several unknown critical factors regarding the pathogenesis of coinfection. From a coinfection model, in which an *S. enterica* serovar Typhi (S_Typhi) was chosen to challenge mice that had been infected 24 h earlier with *Plasmodium berghei* ANKA (*P.b*_ANKA), we evaluated the influence of malaria on cytokine levels, the functional activity of femoral bone marrow-derived macrophages and neutrophils, and intestinal permeability. The cytokine profile over eight days of coinfection showed exacerbation in the cytokines MCP-1, IFNγ and TNFα in relation to the increase seen in animals with malaria. The cytokine profile was associated with a considerably reduced neutrophil and macrophage count and a prominent dysfunction, especially in ex vivo neutrophils in coinfected mice, though without bacterial modulation that could influence the invasion capacity of ex vivo S_Typhi obtained from liver macerate in non-phagocyte cells. Finally, irregularities in the integrity of intestinal tissue evidenced ruptures in the enterocyte layer, a presence of mononuclear leukocytes in the enterocyte layer, an increase of goblet cells in the enterocyte layer and a high volume of leukocyte infiltrate in the sub-mucosa were greatly increased in coinfected animals. Increases of mononuclear leukocytes in the enterocyte layer and volume of leukocyte infiltrate in the sub-mucosa were also seen in monoinfected animals with *P. berghei* ANKA. Our findings suggest malaria causes a disarrangement of intestinal homeostasis, exacerbation of proinflammatory cytokines and dysfunction in neutrophils that render the host susceptible to bacteremia by *Salmonella* spp.

## Introduction

Bacteremia caused by *Salmonella enterica* (typhoid or non-typhoid) in malaria patients is a major public health concern in many Africa countries^1–8^. To date, numerous mechanisms have been proposed to explain susceptibility to malaria-induced *Salmonella*^6,9–14^, and hemolysis caused by *Plasmodium* sp. causes the impairment of a variety of host defense mechanisms. Pioneering studies have already associated macrophage and neutrophil dysfunctions as having an important role in increasing susceptibility to *Salmonella* bacteremia, since these cells are one of the main phagocytes of bacteria^2,4,6,8,12,13,15^. The hemolysis caused by *Plasmodium* sp. induces the secretion of the enzyme heme oxygenase 1 (HO-1) that efficiently ensures the iron recycling and, at the same time, prevents the harmful pro-oxidant effects of the heme group. As a result, malarial hemolysis impairs the ability to develop neutrophils in order to mount a competent oxidative burst so that they become niches for *Salmonella* and other Gram-negative bacterial co-infections^6,12,13,16,17^. In the case of macrophage, malaria hemolysis also affects macrophage iron homeostasis. The iron liberated from heme by HO-1 enters macrophages and, since iron is an essential micronutrient for the replication of *Salmonella* spp. and other intracellular pathogens, macrophage iron retention in malaria favors bacterial multiplication^6,16,17^. In addition, macrophage iron contents enhance cytokine dysregulation and lead to the infiltration of monocytes and neutrophils in the lamina propria, which are hallmarks of pathology in bacteremia for *Salmonella* or other invasive bacteria in malaria infection^11,16^.

Malaria infection also affects intestinal permeability and facilitates invasions of enteric pathogenic bacteria^18–20^. One clinical study addressed intestinal permeability in patients with falciparum malaria, and sucrose, lactulose and mannitol in urine were used as an indication of alterations of this permeability^18^. There are still several critical factors that are unknown regarding the pathogenesis of *Plasmodium-Salmonella* coinfection. Several models have proposed plausible epidemiological situations in malaria endemic areas without basic sanitation. These situations involve simultaneous infections or infections occurring in the days after the malarial infection^10,12–14,21^. Thus, a *Plasmodium-Salmonella* coinfection model on which oral bacterial challenge happened at the beginning of the rodent malaria simulation can provide insights into how parasitemia compatible to *P. falciparum* malaria promotes the susceptibility to bacteremia. Herein, a coinfection model was established challenging *Pb*_ANKA-infected BALB/c mice with a serovar S_Typhi. Serum cytokines, functional phagocytic activity and histopathological-morphometric analyses of the intestinal mucosa were compared with groups of monoinfected mice with *P.b*_ANKA or S_Typhi in order to aid the understanding of these factors on susceptibility to septicemia during malaria.

## Results

### Susceptibility of mice with malaria to Salmonella enterica sv Typhi infection

**Figure 1:**
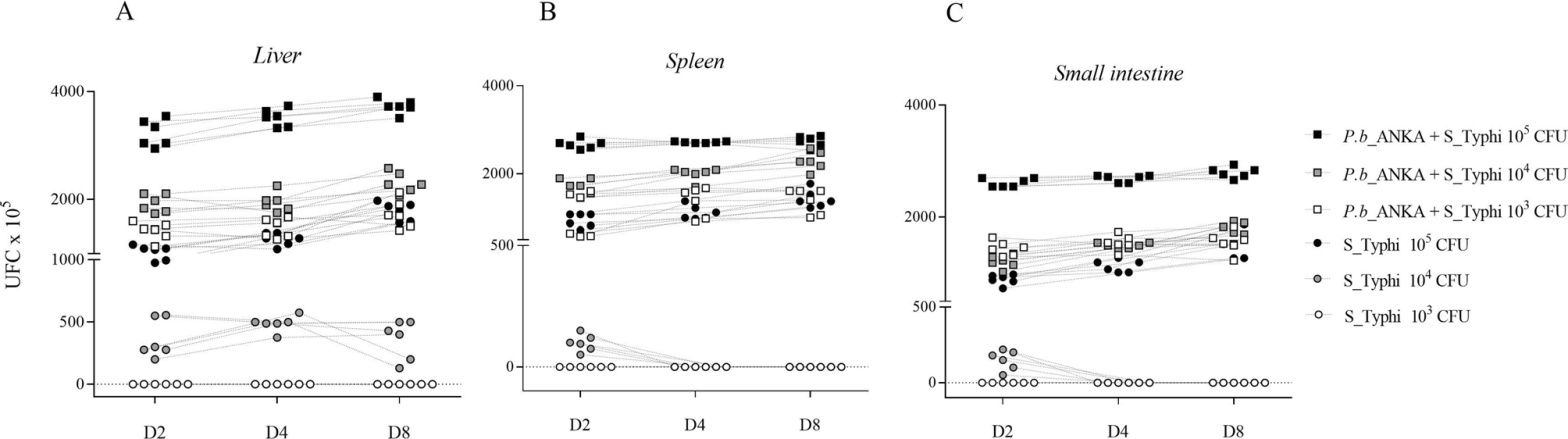
*Plasmodium berghei* ANKA and *Salmonella enterica* serovar Typhi coinfection model. In the malaria infection model developed, three groups of animals received an intraperitoneal dose of 10^6^ erythrocytes infected with *P. berghei* ANKA on D0, and challenged with S_Typhi on D1. Group 1 received 10^5^ S_Typhi CFUs on D1 (Pb + S_Typhi 10^5^). Group 2 received 10^4^ S_Typhi CFUs on D1 (Pb + S_Typhi 10^4^), and Group 3 received 10^3^ S_Typhi CFUs (Pb + S_ Typhi 10^3^). Three other groups were challenged with only S_Typhi on D1, Group 4 (S_Typhi 10^5^), Group 5 (S_Typhi 10^4^) and Group 6 (S_Typhi 10^3^). The animals were euthanized for the quantification of CFUs of the liver, spleen and intestine in SS-Agar at 37 °C. This experiment was replicated once.

Most *Salmonella enterica* serovares (sv) can cause intestinal infections. For the selection of the bacterial strain to be used in the coinfection model, the three following strains were used: *Salmonella enterica* serovar Typhi (S_Typhi), *Salmonella enterica* serovar Chloreaesius (S_Chloreaesius) and *Salmonella enterica* serovar Salamae (S_Salamae) (Figure S1). Only the *S. enterica* serovar Typhi managed to evolve in the invasion, and increased the number of colonies established in 48 h. Based on the hypothesis that malaria infection would act as a susceptibility factor to *Salmonella* invasion, the experimental design of coinfection was tested in order to define the minimum bacterial concentration in which bacteremia occurred only in mice previously infected with malaria, and not in healthy mice. The *Plasmodium*-*Salmonella* coinfection model was established by comparing three different doses of S_Typhi (1 × 10^5^, 1 × 10^4^ and 1 × 10^3^ CFU). For comparison, another three groups of S_Typhi monoinfected mice received only 1 × 10^5^, 1 × 10^4^ 1 × 10^3^ CFU, respectively. The bacterial culture was positive in all coinfection plates, on all sampled days (D2, D4 and D8), while the group that was monoinfected with *S. enterica* sv Typhi infected with 1 × 10^3^ CFU did not show bacterial growth in any of the macerated organs on any of the analyzed days. The presence of colony-forming units in the macerate of the liver, spleen and intestine was only found in the respective *Plasmodium-Salmonella*-coinfection group (Fig. 1A–C). The results allowed us to determine that the inoculum of 10^3^ CFU of *S. enterica* sv Typhi was only able to develop bacteremia in animals previously infected with malaria. The parasitemia of *P. berghei* ANKA in *Plasmodium-Salmonella*-coinfected mice on days D2, D4 and D8 did not differ from those of malaria-monoinfected mice (Figure S2).

### Plasma cytokine profile during malaria and *Salmonella enterica* sv Typhi coinfection in relation to malaria- and Salmonella- monoinfections

**Figure 2:**
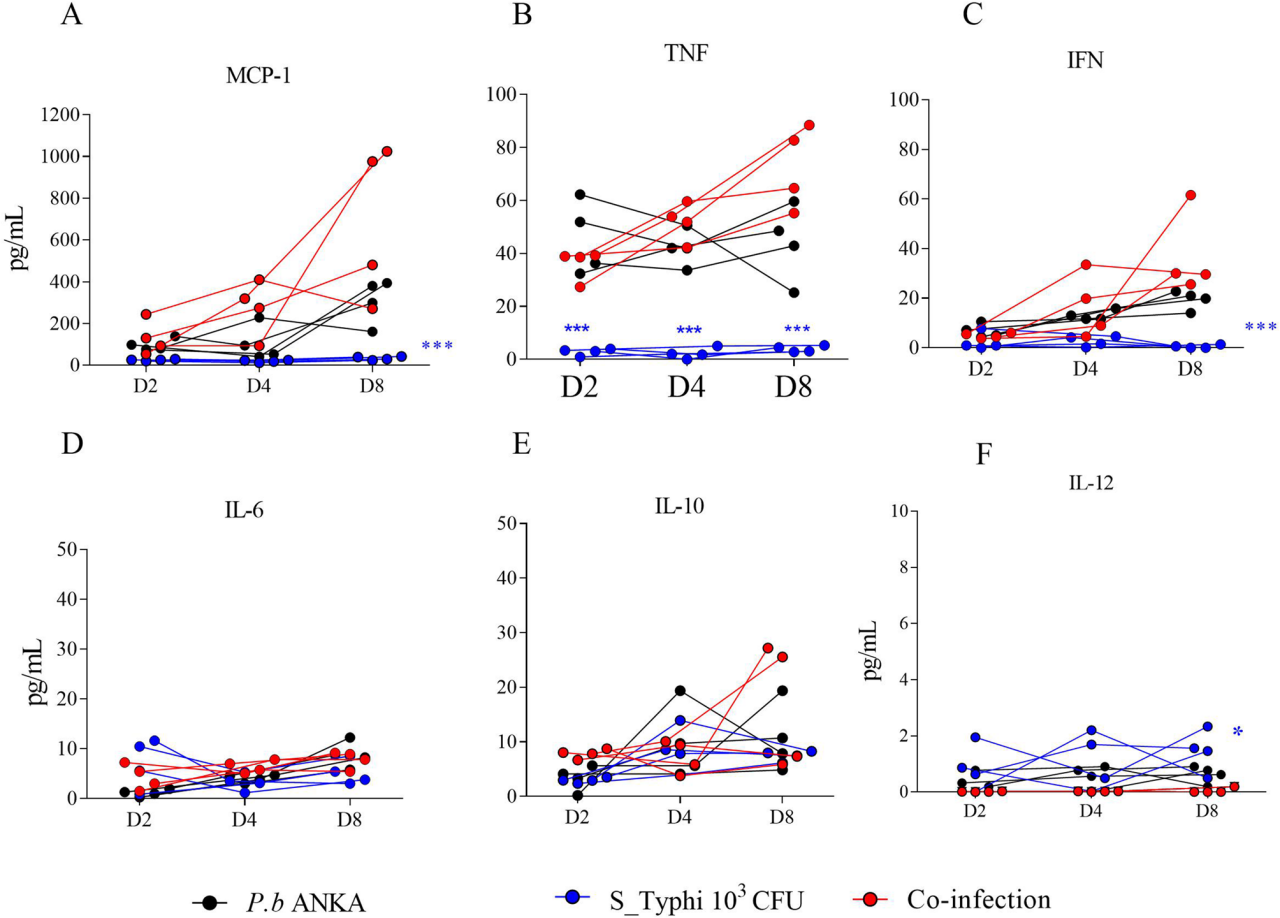
Inflammation response on days D2, D4 and D8 in the coinfection model. The plasma of four animals was sampled for the determination of inflammation response using a cytometric bead array (Mouse Inflammation CBA Kit; BD-Biosciences/USA). (**A**) MCP-1; (**B**) TNFα; (**C**) IFNγ; (**D**) IL-6; (**E**) IL-10 and (**F**) IL-12. The data for cytokines and chemokines are grouped by mean and standard deviation in a spreadsheet using the GraphPad Prism program (version 7). Asterisks: level of significance of *p < 0.05, **p < 0.005 and ***p < 0.0005.

Since production of inflammatory cytokines and chemokines are hallmarks of pathology in bacteremia for *Salmonella* or other invasive bacteria in malaria infection, the inflammation response was evaluated using a cytometric bead array (CBA Mouse Inflammation Kit; BD-Biosciences/USA). MCP-1, TNFα and IFNγ were the most secreted followed by IL-6, IL-10 and IL-12 (Fig. 2). Both *Pb*_ANKA-infected BALB/c mice and coinfected mice showed exacerbation in the cytokines MCP-1, TNFα and IFNγ, which may contribute to the susceptibility of the organism to *Salmonella* invasion. On the other hand, no differences were found with IL-6 and IL-10 between groups, while a slight increase in IL-12 was observed with animals with S_Typhi monoinfection. Nonetheless, these data presented inconclusive results.

### Evaluation of phagocytic activity in medullary subpopulations

**Figure 3:**
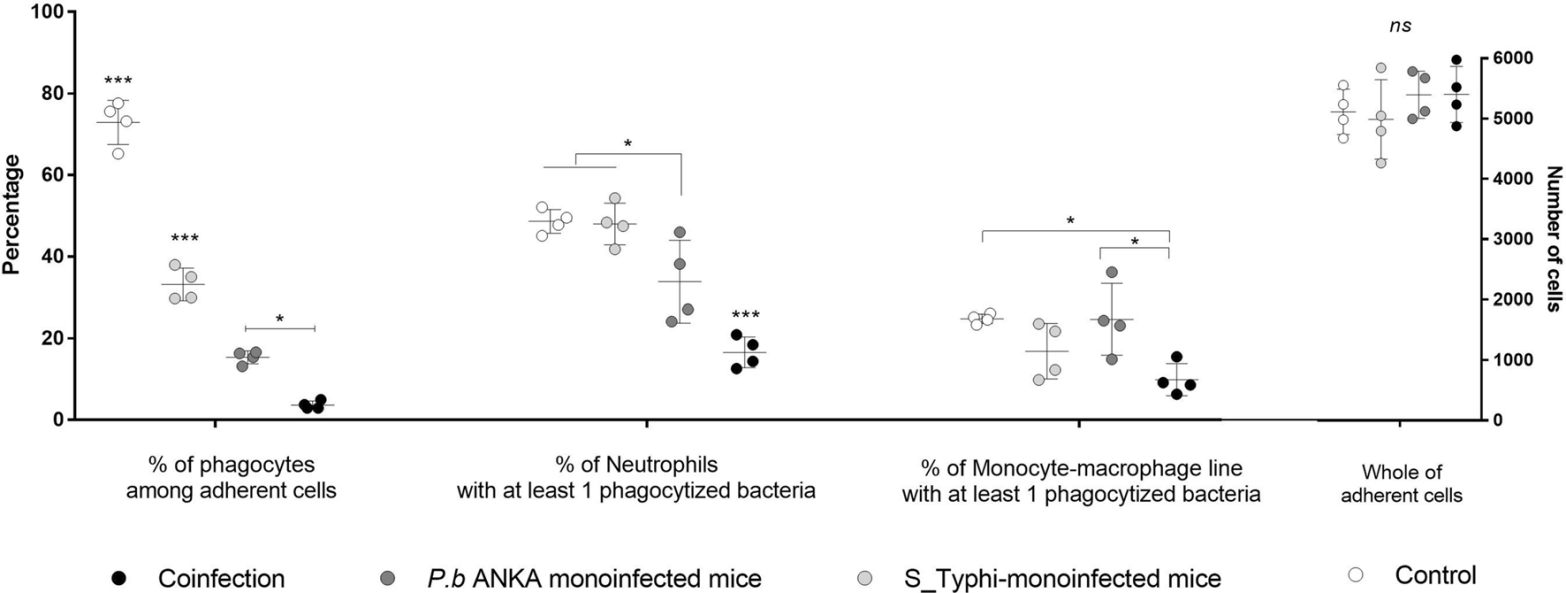
Evaluation of phagocytic activity of ex vivo medullar neutrophils and macrophages. After 24 h of ex vivo incubation on glass slides of cells recovered from the femur of anesthetized mice from coinfected, monoinfected groups with *P. berghei* ANKA or S_Typhi and control group. After washing, the adherent cells were incubated with Phorbol 12-myristate 13-acetate (PMA) for activation in the phagocytosis assay. A suspension of S_Typhi killed by formalization was incubated with the adherent cells. Finally, the slides were washed, fixed in picric acid and stained using the Romanowsky method. Neutrophils and monocytes were visualized through staining characteristics and the total number of phagocytic bacteria was calculated. The phagocytic activity in cells between groups was compared using the percentage of phagocytes between adherent cells; % of neutrophils and % of macrophage containing phagocytic bacteria, respectively. The phagocytic activity in cells was compared by multiple comparison by Tukey’s method. The data are shown in a scattered plot with mean and standard deviations. Asterisks: level of significance of *p < 0.05, **p < 0.005 and ***p < 0.0005.

The study of susceptibility to bacterial coinfected in malaria patients has focused on dysfunction of phagocytic cells macrophages and neutrophils. Our study evaluated the phagocytic activity of ex vivo, PMA-activated, adherent, medullary subpopulation cells against a suspension of 10^3^ CFU of formalin-inactivated S_Typhi (see the self-explanatory diagram of the ex vivo phagocytic assay in Figure S3). The total number of adherent cells showed no differences in all groups Fig. 3). Coinfected mice showed a very low percentage of (neutrophil and macrophage) phagocytes cells based on their characteristics under Romanowsky’s staining method when compared to other groups. The percentage of neutrophils with at least one phagocyted bacteria were more greatly affected in coinfected mice in relation to other groups, including in *Pb*_ANKA-infected BALB/c mice which was reduced in relation to the control mice. The percentage of monocytes with at least one phagocyted bacteria was also affected in coinfected mice, but it was possible though to note that ex vivo neutrophils showed a prominent phagocytic dysfunction.

### Evaluation of the presence of bacterial modulation

**Figure 4:**
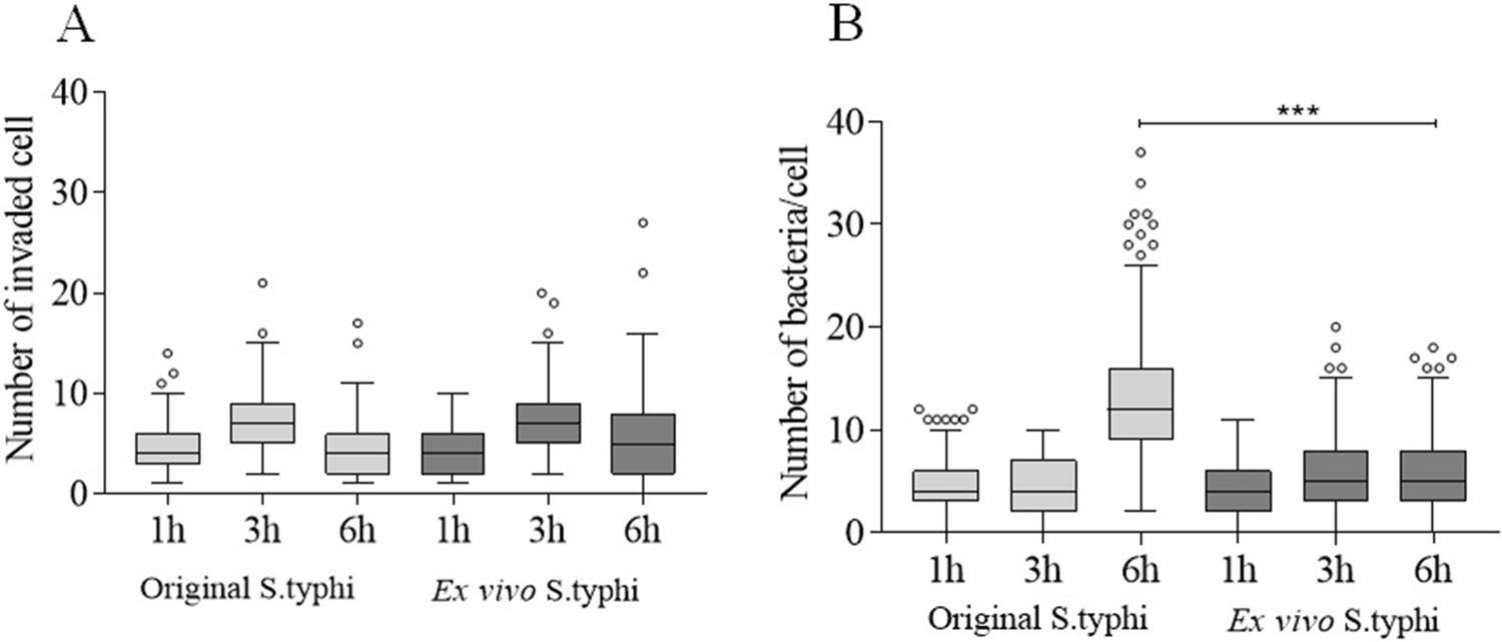
Comparison of the invasive capacity of pre-inoculum and ex vivo obtained bacteria. HeLa cell invasion assay to evaluate the possibility of ex vivo *Salmonella* having its phagocytic activity influenced by some modulation due to host response. (**A**) Number of cells invaded and (**B**) number of bacteria per cell between aliquots of S_Typhi strain used in pre-inoculum conditions with ex vivo strain obtained from liver macerate. Pre-inoculum aliquot was obtained after growth of nine hours in LB broth and stored at − 80 °C for more than 15 days until the moment of the assay. The ex vivo aliquot was obtained from a macerated mouse liver coinfected on D4 and frozen at − 80 °C. The two ex vivo and pre-inoculum bacteria were thawed and set for invasion assay in HeLa cells. Pre-inoculum bacteria were diluted in a series of 10–10 and sown in SS-Agar medium to define the inoculum equivalence of ex vivo bacteria. The invasion time was 1, 3 and 6 h. After these periods, medium supplemented with gentamicin was added to the culture medium for 1 h in order to eliminate external bacteria. Slides were then washed, stained using the Romanowsky method and bacteria visualized. The data are shown in a box plot with mean and standard deviations. Asterisks: level of significance of *p < 0.05, **p < 0.005 and ***p < 0.0005.

In malaria monoinfection, an intraperitoneal dose with 10^6^ erythrocytes infected with *P. berghei* ANKA on D0 is able to cause BALB/c mice to die around day 20 due to high levels of parasitemia (> 60–70%). Coinfection did not lead to a worse outcome because parasitemia was similar to that of *P. berghei* ANKA-monoinfected mice, when the animals were euthanized around twenty days (data not shown). It was also investigated whether the strain of S*_*Typhi recovered from the livers of coinfected mice underwent some modulation that could influence the invasion capacity in non-phagocyte cells, whereas bacterial pathogens tightly regulate the expression of virulence genes in response to very specific conditions. The HeLa cell invasion assay was used to compare the S_Typhi strain used in pre-inoculum conditions with the ex vivo strain obtained from liver macerate (see the self-explanatory diagram of invasion assay in Figure S4). Both behaved similarly in relation to the amount of invaded cells (Fig. 4A), however, the pre-inoculum invaded more HeLa cells after six hours, which indicates that the post-invasion bacterium did not undergo any modulation that made it more invasive (Fig. 4B).

### Histopathological analysis by photomicroscopy

**Figure 5:**
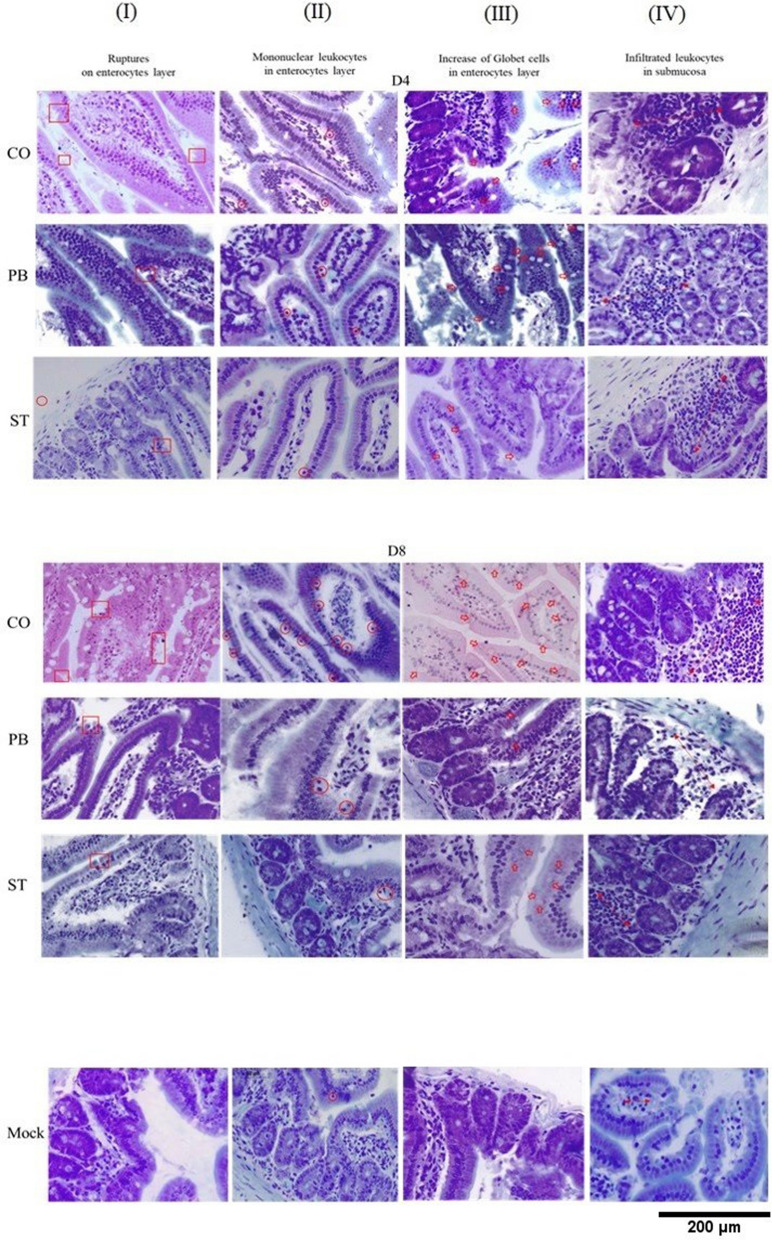
Irregularities in the small intestines of BALB/c mice caused by coinfection by *Plasmodium berghei* ANKA and *Salmonella enterica* serovar Typhi. Four types of changes were evidenced in photomicrographs and highlighted in columns I to IV. (**I**) appearance of ruptures in the enterocyte layer, (**II**) presence of mononuclear leukocytes in the enterocyte layer, (**III**) increase of goblet cells in the enterocyte layer and (**IV**) volume of leukocyte infiltrate in the sub-mucosa. Group CO (coinfection): mice infected with 1 × 10^6^ erythrocytes infected with *P. berghei* ANKA inoculated intraperitoneally on D0, and on D1 the animals were challenged with 1 × 10^3^ CFU of *Salmonella enterica* sp. Typhi by gavage. Group PB: monoinfected mice with 1 × 10^6^ erythrocytes infected with *P. berghei* ANKA intraperitoneally on D0. Group ST: monoinfected mice with 1 × 10^3^ CFU of *Salmonella enterica* sp. Typhi inoculated via gavage on D1. Photomicrographs of groups CO, PB and ST distributed in columns I to IV between D4 and D8. Last sequence of photomicrographs with images of control animals. Column I: presentation of ruptures at the border of the villus (red square); column II: infiltrate of mononuclear leukocytes in the enterocyte layer (red circle); column III: highlight of goblet cells (red broad arrows); column IV: dimension of the volume of leukocyte infiltrate in the mucosa region (red arrow at both ends). Bar 200 μm.

**Figure 6:**
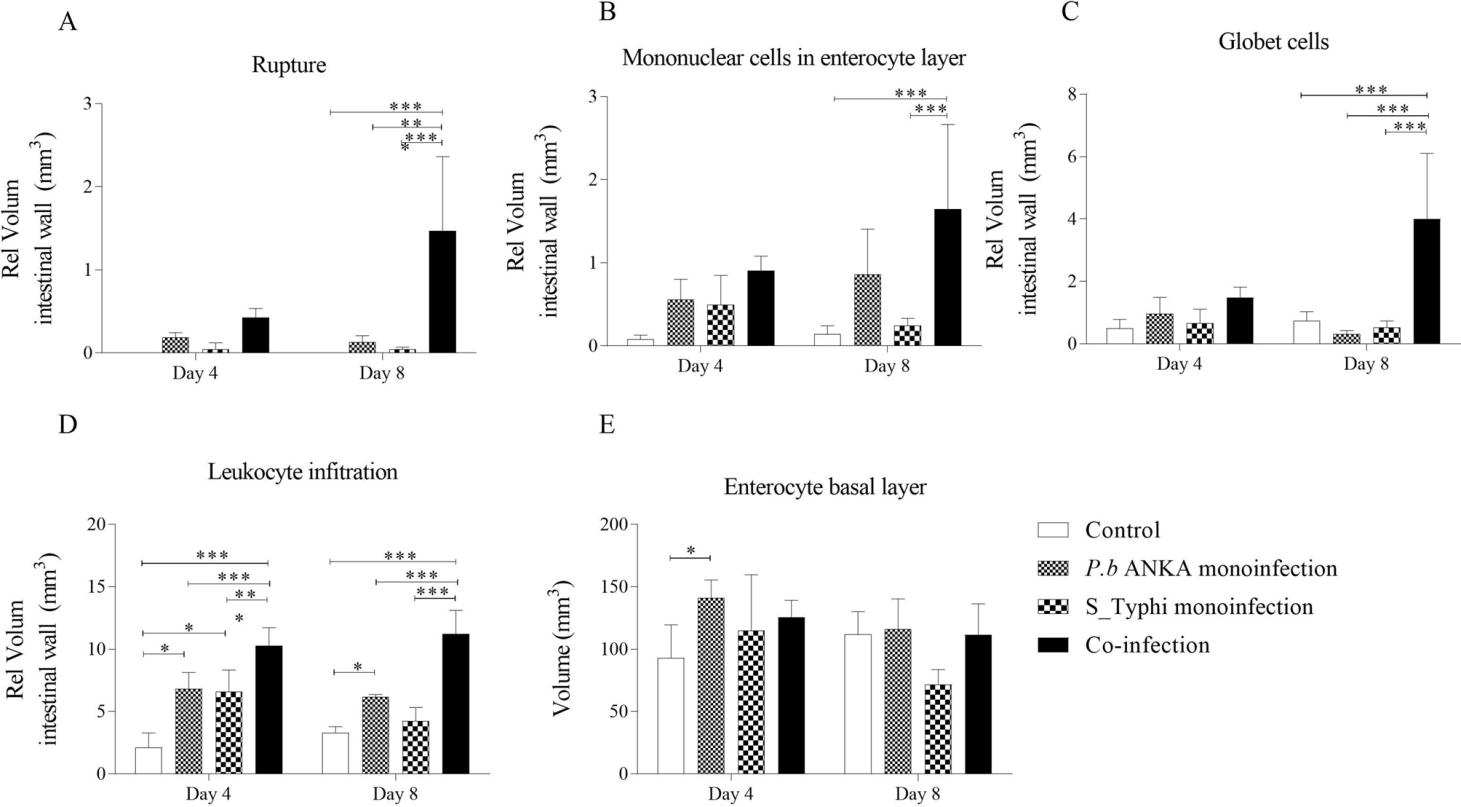
Quantification of irregularities in the small intestines caused by coinfection by *Plasmodium berghei* ANKA and *Salmonella enterica* serovar Typhi. Multiple comparison by Tukey test among the four types of changes evidenced in photomicrographs in coinfection *Plasmodium berghei* ANKA and *Salmonella enterica* serovar Typhi. (**A**) Appearance of ruptures in the enterocyte layer, (**B**) Presence of mononuclear leukocytes in the enterocyte layer, (**C**) Increase in goblet cells in the enterocyte layer and (**D**) Volume of leukocyte infiltrate in the mucosa. The data are shown in a box plot with mean and standard deviations. Asterisks: level of significance of *p < 0.05, **p < 0.005 and ***p < 0.0005.

The above results showed that the invasion by S_Typhi was mainly due to the presence of malaria rather than the invasive capacity of the bacterium. To assess whether malaria infection affects intestinal tissue integrity or irregularities on mucosa sublayers are a result of coinfection, histopathological sections were submitted to morphometric analysis. The volume and intestinal wall did not differ between the groups following the quantification by Cavalieri technique, nor did the submucosal, muscular and serous layers show differences among the groups (data not shown). Morphometric measurements of intestinal volume in the cross sections of histopathological sections increased by magnification 100. The intestinal tissue integrity and irregularities on mucosa sublayers were evaluated by images of sections using a stereomicroscope. Blind analyses were evaluated in longitudinal extensions at magnification 400, where counting systems containing points were superimposed on the images in the program Imod version 4.7/stereology module^22^ (Figure S5). Four types of changes were evident (Fig. 5): (I) appearance of ruptures in the enterocyte layer, (II) presence of mononuclear leukocytes in the enterocyte layer, (III) increase in goblet cells in the enterocyte layer and (IV) increase in volume of leukocyte infiltrate in the sub-mucosa. Irregularities in the intestinal tissues were quantified by the Delesse principle. It was possible to observe ruptures in the enterocyte layer on days D4 and D8 in the three groups (column I, Fig. 5), but events increased in coinfected animals. *Pb_*ANKA monoinfected animals showed a slight increase in ruptures, while the S_Typhi-monoinfected mice (ST) showed very few ruptures, which indicates that these ruptures may be the gateway for the bacterium (Fig. 6A).

The intestinal mucosa showed the presence of mononuclear leukocytes among the enterocytes (column II, Fig. 5). In this case, the three groups presented more than the control group on D4, the mononuclear cells infiltrate was much higher on D8 in the coinfected ones in relation to S_Typhi and control groups, however it did no differ of *P. berghei* ANKA infected mice (Fig. 6B). Regarding the presence of mucoid or goblet cells in the epithelial layer (column III, Fig. 5), only the co-infected animals showed an increase of these cells (Fig. 6C). The volume of leukocyte infiltrate in the mucosa showed the main differences (column IV, Fig. 5) and the three groups differed from the control on the 2 days, while the volume in coinfected animals was higher than both groups which were monoinfected with *P. berghei* ANKA and S_Typhi (Fig. 6D). Interestingly, the volume of leukocyte infiltrate in S_Typhi-infected mice recovered normal volume on D8 that did not differ of control group. In relation to enterocyte basal layer, the volume was inconclusive (Fig. 6E). With this, it is possible to suggest that malaria causes a disruption of intestinal homeostasis and makes it susceptible to *Salmonella* invasion.

## Discussion

Due to the overlap in terms of geographical distribution, children with recent or acute malaria have a higher risk of bacterial infection and death. This knowledge comes from clinical studies according to which the effects of malaria on the immune response against *Salmonella* bacteremia^1,2,7^. The exact nature of this association is still unknown, due to heterogeneity of data and lack of adequate controls corresponding to severity (reviewed by^6^). Thus, little is known about whether malaria affects host resistance to intestinal colonization with typhoidal or non-typhoidal *Salmonella* *enterica* after bacterial ingestion. Herein, we managed to develop a model of coinfection by *Salmonella enterica* serovar Typhi and *P. berghei* ANKA in BALB/c mice, in which animals were already susceptible to *Salmonella* bacteremia after a day of malarial infection. Our model showed that a level of 1% parasitemia and 24 h of intraperitoneal infection was able to make the animals susceptible to invasive salmonellosis. These parasitemias are commonly observed in human malaria, especially in African children. Therefore, the leukocyte hemozoin pigment is a stronger predictor of malaria disease than parasitemia itself, which shows that concomitant infection can mask the diagnosis of salmonellosis infection when antimalarial treatment has still not improved the child's symptoms^9,23^.

Although malaria is an acute systemic infection, it is known that gastrointestinal symptoms, such as nausea, vomiting, abdominal pain and diarrhea, are common during the acute phase of malaria. Nutrient malabsorption has been reported in severe falciparum malaria, suggesting that the environment in the intestinal lumen could be altered^18,24^. In a study on malaria *falciparum*, damage in the intestinal epithelium was evidenced due to permeation of compounds, such as monosaccharides and disaccharides, that are normally relatively impermeable in a healthy intestinal mucosa but in this case they were observed in the urine of these patients^18^. In this context, we performed a thorough analysis of intestinal tissue in the histopathological sections in malaria infections on D8 in order to evaluate changes in intestinal volume and tissue. The volume of the lumen and intestinal wall were not altered between the groups probably because on the eighth day the changes are probably still focal. The intestinal epithelium provides a protective barrier against enteric pathogens and upon disruption increases susceptibility to infections or impairs immunity^25^. One of the main findings observed here was that monoinfection by *P. berghei* ANKA malaria was able to alter the integrity of the epithelial layer of the intestine. These findings are in agreement with altered intestinal epithelium permeability in both *Plasmodium falciparum* infected patients and murine malaria model^10,18,25^. The appearance of ruptures in the enterocyte layer seems a detachment of the intestinal epithelia demonstrated by Taniguchi and cols^10^. Here, ruptures in the enterocyte layer were exacerbated in coinfected animals suggesting that ruptures in the epithelial layer caused by malaria predispose the host to bacteremia^23,26^.

Changes in the architecture of the intestinal tissue included the increase in the number of goblet cells and in the cell density, which in the latter is characterized by volume of the leukocyte infiltrate in the sub-mucosa. The goblet cells are responsible for the layer of mucus that covers the epithelium to facilitate the removal of food content and are also part of the first line of defense against damage caused by pathogenic bacteria and bacterial products^27,28^. Here, coinfected mice showed several of these mucoid cells in the enterocyte layer, which indicates that the innate response to the infection bacteria was active in these animals. However, the disarrangement of intestinal homeostasis caused by malaria may compromise the fight against the *Salmonella* infection, despite abundance of the goblet cells. In relation to cell density, leukocyte infiltrate was greatly increased in coinfected mice. Several coinfection models showed that these infiltrates are composed of inflammatory monocytes and neutrophils^6,9,21,25^. Despite this increase, the phagocytic activity has been one of the most widely-accepted explanations for susceptibility to bacteremia in malaria^2,4,14,15^. Herein, phagocytic dysfunctions of macrophage and neutrophil progenitors in co-infected mice were greatly reduced, which indicates that these effector cells would have already been compromised in the primary lymphoid organs. In addition, hematopoiesis impairment may be associated with factors associated with bacteria, since MCP-1, TNFα and IFNγ levels and parasitemia in co-infected mice did not differ from those monoinfected by *P. berghei* ANKA.

In relation to the bacteria, the survival and growth of salmonellae within host cells are important for bacterial virulence, and many genes that are required for invasion are not expressed when bacteria are grown on rich media^29^. Herein, we demonstrated that phenotypically the ex vivo S_Typhi recovered from the liver of coinfected animals did not become more invasive than pre-inoculum bacteria. One study showed that animals previously infected with *P. yoelii* exhibited intensified colonization of a noninvasive, commensal, *Escherichia coli* strain, which suggests that the disturbance of the microbial community by malaria opens up an ecological niche that can be harnessed by pathogenic bacteria^21^. However, we are cautious as to whether our data show that malaria infection makes mice susceptible to invasion of pathogenic bacteria, despite some modulation in vivo, or if, phenotypically, the S_Typhi grown in rich media would already be able to invade the host cells^9,14^.

One limitation of the study was to use a serovar Typhi from the *Salmonella enterica* line, since pediatric bacteremia caused by *P. falciparum* infection mainly involves non-typhoid invasive *Salmonella*. In addition, the models of coinfection or monoinfection in mice use *S. enterica* serovar Typhimurium because it spreads systemically in this host^9,12,14,21,30^. From three serovars of *Salmonella enterica,* we defined a dose with S_Typhi that managed to evolve in the invasion of BALB/c mice only in those previously infected with malaria. Other limitation was that in our model, the host was challenged with the bacterium the day after malaria infection, while children who develop malaria-*Salmonella* coinfection are probably already colonized by these pathogenic bacteria^15^. Although, that is assumption that *Salmonella* infection is subsequent to malaria, i.e. it is reasoned that in endemic areas individuals would be most likely to become infected with typhoidal or non-typhoidal *Salmonella* after contracting malaria^9^. The number of mice used here seemed another limitation, however they were sufficient to demonstrate the major differences. Despite these limitations, our model provided us with a new view on intestinal mucosal alteration that predisposed BALB/c mice to bacteremia due to malaria.

In conclusion, our findings provide descriptive insights into how mucosal responses to a bacterial pathogen are altered in a simultaneous malaria infection. Although the knowledge of malaria as a susceptibility factor to *Salmonella* bacteremia is evident, the pathophysiological mechanisms are still not well understood due to their complexity and lack of well-established experimental models. This study aimed to establish an experimental model and evaluate the pathophysiology with better coverage of intestinal histopathology, systemic immune factors and functional assays of phagocytic dysfunction. The inflammatory exacerbation evidenced by the increase of cytokines and chemokines with the dysfunction especially of neutrophils is also well known and was well characterized as an effect of an exacerbated inflammatory response. Finally, our main findings were the alteration in the epithelial integrity of the intestine caused by monoinfection with *P. berghei,* which was identified as a possible cause of susceptibility to *Salmonella* invasion. Knowledge on the influence on intestinal tissue and the interaction of effector immune mechanisms in *Salmonella*-malaria coinfection is absolutely essential for creating strategies to combat the bacterial infections in affected populations.

## Material and methods

### Strains

Currently, the *Salmonella* nomenclature system defines the genus as being composed of three species: *S. subterranea, S. bongori* and *S. enterica*, the latter having more than two thousand serotypes and, as such, it is classified as a subspecies^31^. The *Salmonella enterica* serovar Typhi (S_Typhi), *Salmonella enterica* serovar Chloreaesius (S_Chloreaesius) and *Salmonella enterica* serovar Salamae (S_Salamae) strains were provided by the Laboratory of Diagnosis and Control of Amazonian Infectious Diseases, Instituto Leonidas e Maria Deane-ILMD/Fiocruz Amazonia. The *Plasmodium berghei* ANKA-GFP strain (clone 15cy1) was conceded by Dr. Claudio Marinho of the Laboratory of Experimental Immunoparasitology, University of São Paulo (USP). The infected red blood cells (iRBC) used in experimental infections were obtained after in vivo passage in BALB/c mice. For infections, aliquots containing 5 × 10^6^ infected red blood cells (iRBCs)/100 µl were stored at − 80 °C were inoculated intraperitoneal (i.p.) on day 0. The controls treated with this simulation were injected with an equal volume of isotonic and neutral phosphate buffer. The parasitemia was performed on days 2, 4 and 8 after analysis using flow cytometry.

### Animals

Female BALB/c mice aged between 6 and 8 weeks were used. In all experiments, the welfare of the animals was taken into consideration. The mice were housed in a standard polycarbonate cage with wood shavings bedding, with a maximum of 5 mice per cage. We also attempted to reduce the stress of individual housing (when necessary) by environmental enrichment with nestlets and small play tunnels. The animal room was kept at a controlled temperature and humidity, with a light and dark cycle of 12 h. Mice had ad libitum access to food and water.

### Anesthesia and euthanasia

All efforts were made to prevent undue stress or pain to the mice. The mice were humanely euthanized once they showed the following clinical signs: lethargy; hypothermia and/or difficulty of breathing. The mice were euthanized with ketamine (300 mg/kg) (Vetbrands, Brazil) and xylazine (22.5 mg/kg) (Syntec, Brazil), and consciousness was checked by testing the foot reflexes, heartbeats and breathing movements. All experiments were performed in accordance with the ethical guidelines for experiments with mice, and the protocols were approved by the National Council for the Control of Animal Experimentation, National Institute of Amazonian Research – INPA (CEUA nº 2018016/2019). The guidelines for animal use and care were based on the standards established by the Brazilian College of Animal Experimentation (COBEA).

### Infection

Initially, one *Salmonella enterica* serovar was selected to be used in the coinfection model with the *Plasmodium berghei* ANKA. The strategy was to select the most invasive of the three bacteria (S_Typhi, S_Chloreaesius and S_Salamae) i.e., the one that presented the most colony forming units (CFU) in the liver, spleen and intestine at 24 h and 48 h post infection. The animals were divided into three groups containing four animals to be individually challenged with the three different serovares. The time necessary to reach maximum exponential growth that was ideal for infection was defined as nine hours of growth in LB broth, and the oral inoculation was performed by gavage technique with a concentration of 1 × 10^9^ CFU in 100 µl in each individual. After euthanasia and removal of organs, they were homogenized, diluted and sown in serial dilution in *Salmonella*—*Shigella* agar culture medium (SS-Agar) and incubated at 37 °C. After 24 h, the colony forming units (CFU) were quantified.

### Establishment of *Plasmodium berghei* ANKA and Salmonella enterica serovar Typhi coinfection model

For establishment of *Plasmodium-Salmonella* coinfection model, three groups of BALB/c mice received an intraperitoneal dose of cryopreserved aliquots containing 5 × 10^6^ erythrocytes infected with *P. berghei* ANKA on day 0 (D0). One group received a dose of 1 × 10^5^ CFU of S_Typhi, the second received 1 × 10^4^ CFU and the third 1 × 10^3^ CFU on D1, by gavage. For the comparison, three other groups containing four mice with monoinfection by S_Typhi, received, a dose of 1 × 10^5^ CFU by gavage on D1. The second received 1 × 10^4^ CFU and the third 1 × 10^3^ CFU, by the same means. The experimental design of malaria-*Salmonella* coinfection was evaluated by the presence and/or absence of colony-forming units in the macerate of the liver, spleen and intestine obtained on D2, D4 and D8. After euthanasia, the organs were removed whole, homogenized, diluted and sown in serial dilution in *Salmonella*—*Shigella* agar culture medium (SS-Agar) and incubated at 37 °C. The group in which S*_*Typhi monoinfected mice did not show bacterial growth in any of the macerated organs on any of the analyzed days was chosen. Parasitemia of *P. berghei* ANKA groups was monitored by flow cytometry.

### Plasmodium berghei ANKA and Salmonella enterica serovar Typhi coinfection model

Three groups containing four mice were classified as S_Typhi monoinfection, *P. berghei* ANKA monoinfection and *Plasmodium-Salmonella*-coinfection. The *P. berghei* ANKA monoinfection and *Plasmodium-Salmonella*-coinfection groups received an intraperitoneal dose of cryopreserved aliquots containing 5 × 10^6^ erythrocytes infected with *P. berghei* ANKA on day 0 (D0). The controls and S_Typhi monoinfected mice received an equal volume of isotonic and neutral phosphate buffer. The determination of parasitemia was performed by flow cytometry. The controls and S_Typhi monoinfected mice received an equal volume of isotonic and neutral phosphate buffer. The animals were submitted to the *Plasmodium*-*Salmonella* coinfection and S_Typhi monoinfection mice received 100 µL S_Typhi 10^3^ in 24 h LB broth via gavage one day after the malarial infection (D1). Mice of control and *P. berghei* ANKA monoinfection groups received an equal volume of isotonic and neutral phosphate buffer. Parasitemia of *P. berghei* ANKA monoinfection and *Plasmodium-Salmonella*-coinfection groups was performed by flow cytometry. Extraction from the intestine occurred on days four and eight (D4 and D8) after malarial infection. The animals were euthanized for the quantification of CFUs in the liver, spleen and intestine, and the homogenized ones were sown in SS-Agar at 37 °C for quantification of CFU.

### Evaluation of functional activity

The phagocytic activity of neutrophils and macrophages was evaluated using cell culture after incubation, which was based on the literature and optimized for the methodology (See the diagram in Figure S3). Briefly, after induction of anesthesia and analgesia, the animals were sacrificed for the removal of medullary subpopulations from the femur on D8. The recovered cells were plated on glass slides (Knittel, Brazil) in DMEM 24-well plates at 37 °C, 5% CO_2_ for panning of adherent cells. After 24 h of incubation, nonadherent cells were removed after three cycles of washing in DMEM medium. The adherent cells were incubated with Phorbol 12-myristate 13-acetate (PMA) for 1 h for activation, and then washed three times in DMEM medium. A suspension of 10^3^ CFU of formalin inactivated S_Typhi that had been treated previously with 37% formalin for 1-h and washed 4 times with neutral phosphate buffer was applied for 3 h at 37 °C, 5% CO_2_. Finally, the slides were washed, fixed in picric acid and stained using the Romanowsky method. Neutrophils were differentiated by the “busy” aspect of nucleus showing several lobes, while cells characterized by horseshoe-shaped nucleus with dishwater-gray cytoplasm and a few tiny granules were characterized as monocyte-macrophage lines. The quantity of phagocytic activity in cells was compared by multiple comparison by Tukey’s method.

### Bacterial invasion assay in HeLa cells

The possibility of ex vivo *Salmonella* strains having been modulated after infection and influencing phagocytic activity was analyzed. For this, the livers of mice that were coinfected on D4 were macerated and frozen at − 80 °C. For comparison, an aliquot of S_Typhi was used under the same conditions as the pre-inoculum (in growth phase of nine hours in LB broth) and stored at − 80 °C for 15 days until the moment of the assay (Figure S4). HeLa cells were grown in 24-well plates containing glass slides (Knittel, Brazil) in DMEM medium with 10% fetal bovine (Sigma, Brazil) at 5% CO_2_ at 37 °C until total confluence. The two ex vivo and pre-inoculum bacteria were thawed and set for the invasion assay in HeLa cells. Pre-inoculum bacteria were diluted in a series of 10–10 and sown in SS-Agar medium to define the inoculum equivalence of ex vivo bacteria. The invasion time was 1, 3 and 6 h. After these periods, medium supplemented with gentamicin was added to the culture medium for 1 h in order to eliminate external bacteria. Slides were then washed, stained using Romanowsky staining and bacteria were visualized.

### Analysis of serum cytokines

Cytokines were measured on D2, D4 and D8 for each of the infection groups. The plasma from four mice was collected for the determination of cytokines using cytometric bead array for mouse inflammation factors (CBA Mouse Inflammation Kit; BD-Biosciences/USA).

### Histopathological evaluation of the intestine

After euthanasia, the intestines of the animals were removed and kept in buffered formalin for 48 h at room temperature. The samples were processed in the Quantitative Morphology Laboratory (LaMiq/UFAM). For this, the intestines were dehydrated in increasing concentrations of 70 and 96% ethanol (2 h in each concentration), pre-infiltrated in 96% ethanol + hydroxyethyl methacrylate plastic historesin solution (Technovit 7100, Külzer-Heraues, Germany) overnight and infiltrated in 100% resin. The samples were arranged in individual Histobloc teflon molds (Külzer-Heraues, Germany) set in plastic resin + polymerizing solution. The molds were heated in an oven at 37 °C until complete polymerization. The analysis of epithelial integrity of the intestine and morphometric measurements were made using stereology of the Cavalieri principle of volume and the Delesse principle, both operated using the Imod program (see Supplemental Material 1).

### Statistical analysis

The prism 5.0 statistical program (GraphPad Software, Inc. USA) was used for the statistical and graphical analysis of this study. The independent variables (treatments) were tested for their normality by the Kolmogorov–Smirnov test. An ANOVA test using a multiple Tukey comparison was used for the following assays: (1) cytokines on days D4 and D8, (2) phagocytosis assays on D8, and (3) S_Typhi invasion of pre-inoculum and ex vivo in HeLa cells. In relation to histopathological analyses, on average 20 serial sections of the intestine were calculated by appropriate equations developed for stereological analyses^22,32,33^. An error coefficient of 5% and deviation of 15% were considered acceptable. The estimate of the volume was determined according to Cavalieri’s principle, and the results were analyzed using one-way ANOVA (see Supplementary material).

## Supplementary Information

Supplementary Information.
