# Author Correction: Events associated with susceptibility to invasive *Salmonella enterica* serovar Typhi in BALB/c mice previously infected with *Plasmodium berghei* ANKA

**DOI:** 10.1038/s41598-023-28888-3

**Published:** 2023-01-31

**Authors:** Yasmin Cabral Moreira, Maele Jordão, Oscar Tadeu Ferreira da Costa, Elizangela Farias, Alysson Guimaraes Costa, Viviane de Farias, Dorval Antonio Mafra Coimbra, Tatiana Bacry Cardoza, Yury Oliveira Chaves, Patricia Puccinelli Orlandi, Fabio Trindade Maranhão Costa, Paulo Afonso Nogueira

**Affiliations:** 1 Fundação Oswaldo Cruz-Fiocruz, Instituto Leônidas e Maria Deane (ILMD- Fiocruz Amazônia), Manaus, AM Brazil; 2 Programa de Pós-Graduação em Biologia da Interação Patógeno Hospedeiro, ILMD- Fiocruz Amazônia, Manaus, AM Brazil; 3 Instituto de Ciências Biológicas, Universidade Federal do Amazonas, Manaus, AM Brazil; 4 Diretoria de Ensino e Pesquisa, Fundação Hospitalar de Hematologia e Hemoterapia do Amazonas, Manaus, AM Brazil; 5 Fundação de Medicina Tropical Dr Heitor Vieira Dourado, Instituto de Pesquisa Clínica Carlos Borborema, Manaus, AM Brazil; 6 Programa de Pós-Graduação em Imunologia Básica e Aplicada, Universidade Federal do Amazonas, Manaus, AM Brazil; 7 Fundação Oswaldo Cruz-Fiocruz, Programa de Pós-graduação Stricto sensu em Biologia Parasitária do Instituto Oswaldo Cruz, Rio de Janeiro, RJ Brazil; 8 Laboratório de Doenças Tropicais, Instituto de Biologia, Universidade Estadual de Campinas, Campinas, SP Brazil

Correction to: *Scientific Reports* 10.1038/s41598-021-82330-0, published online 1 February 2021

The Funding section in the original version of this Article was omitted. The Funding section now reads:

“This work was supported by Fundação de Amparo à Pesquisa do Estado do Amazonas (FAPEAM; EDITAL N. 005/2019 - PAPAC)”.

The original Article has been corrected.

