# Supplementary material for: Events associated with susceptibility to invasive Salmonella enterica serovar Typhi in BALB/c mice previously infected with Plasmodium berghei ANKA
Source: Sci Rep. 2021 Feb 1;11:2730. doi: 10.1038/s41598-021-82330-0 (PMC7851127; doi:10.1038/s41598-021-82330-0)
Supplement: Supplementary file 1 — Supplementary Information. [file 41598_2021_82330_MOESM1_ESM.docx]

**Events associated with susceptibility to invasive *Salmonella enterica* serovar Typhi in BALB/c mice previously infected with *Plasmodium berghei* ANKA**

Yasmin Moreira ^1,2^*, Maele Jordão^1,2^*, Oscar Tadeu Ferreira da Costa^3^, Elizangela Farias^1^, Alysson Guimaraes Costa^4,5,6^, Viviane de Farias^1^, Dorval Antonio Mafra Coimbra^1^, Tatiana Bacry Cardoza^1^, Yury Oliveira Chaves^1,7^, Patricia Puccinelli Orlandi^1^, Fabio Trindade Maranhão Costa^8^, Paulo Afonso Nogueira^1,2,5,6^

**SUPPLEMENTARY DATA**

Include:

1) **Selection of *Salmonella enterica* serovar for the development of the malaria coinfection model and Figure S1.**

# **2) Monitoring of parasitemia of *P. berghei* ANKA by flow cytometry in *Plasmodium-Salmonella*-coinfected- and malaria-monoinfected mice on days D2, D4 and D8, and Figure S2**

**3) Figure S3: The line diagram for clarifying the rationale of bacterial modulation.**

**4) Figure S4: The line diagram for clarifying the rationale of bacterial modulation.**

**5) Methods for the analysis of epithelial integrity of the intestine based on morphometric measurements using stereology of the Cavalieri principles.**

**6) Figure Supplementary 3. Stereology of the volume of the small intestine and density of the volume of components in the intestinal wall**

# **1) Selection of *Salmonella enterica* serovar for the development of the malaria coinfection model**

Most *Salmonella enterica* serovares (sv) can cause intestinal infections. For the selection of the bacterial strain to be used in the coinfection model, three strains were used: the *Salmonella enterica* serovar Typhi (S_Typhi), *Salmonella enterica* serovar Chloreaesius (S_Chloreaesius) and *Salmonella enterica* serovar Salamae (S_Salamae). Three groups containing 4 BALB/c mice received 100µL per individual and orally (gavage) with 1x10^9^ CFU of S_Typhi, S_Chloreaesius or S_Salamae. The animals did not present any systematic changes in the 48h period of infection. After 24h, two animals from each group were euthanized for removal of liver, spleen and small intestine for the CFU count, and the other two after 48 hours. The invasion was successful with all three bacteria. Only the *S. enterica* serovar Typhi managed to evolve in the invasion, and increased the number of colonies established in 48 h (Figure 1A). The S_Chloreaesius and S_Salamae showed a decrease in the amount of colonies between 24 h and 48 h of infection, demonstrating a possible immune response in the control of bacterial invasion ((Figure S1 B-C)).

#
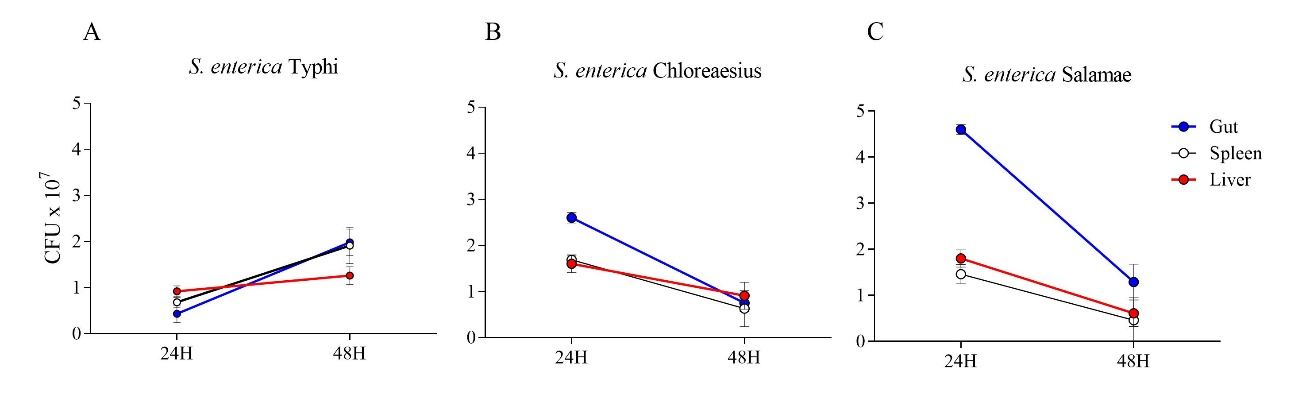


# **Figure S1. Selection of *Salmonella enterica* serovar Typhi for the development of the malaria coinfection model.** For the development of the model, three *Salmonella enterica* serovars were employed using oral inoculation by gavage at a concentration of 1x10^9^ CFU in 100µL. The quantification of colony-forming units (CFUs) in the macerated liver, spleen and intestine of euthanized animals 24 h and 48 h post-challenge are shown. The homogenized ones were sown in SS-Agar at 37 °C for CFU quantification. (A) S_Typhi; (B) S_Chloreaesius and (C) S_Salamae. The animals were euthanized for the quantification of CFUs of the liver, spleen and intestine in SS-Agar at 37 °C.

# **2) Monitoring of parasitemia of *P. berghei* ANKA by flow cytometry in *Plasmodium-Salmonella*-coinfected- and malaria-monoinfected mice on days D2, D4 and D8, and Figure S2**

**Figure S2. Comparison of parasitemia of Pb-ANKA in malaria monoinfected- and Plasmodium-Salmonella-coinfected mice.** After inoculation of cryopreserved aliquots containing 5 × 10^6^ infected red blood cells (iRBCs)/100 microliters of *Plasmodium berghei* ANKA-GFP strain (clone 15cy1) on day 0, the determination of parasitemia was monitored by flow cytometry on days 2, 4 and 8.

**Figure S3. Diagram *ex vivo* phagocytic assay**

**
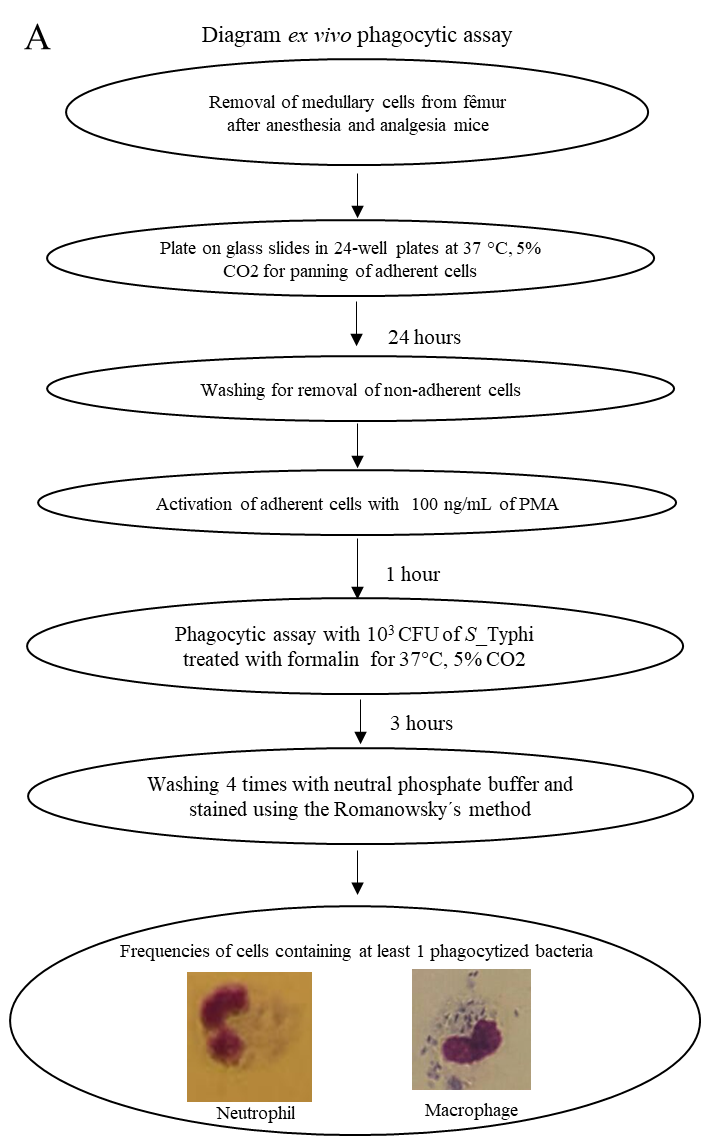
**

**Figure S3. Diagram *ex vivo* phagocytic assay** After induction of anesthesia and analgesia, the animals were sacrificed for the removal of medullary subpopulations from the femur. The recovered cells were plated on glass slides (Knittel, BRA) in DMEM 24-well plates at 37 °C, 5% CO_2_ for panning of adherent cells. After 24 hours of incubation, washing with DMEM medium removed nonadherent cells. The adherent cells were incubated with Phorbol 12-myristate 13-acetate (PMA) during 1 hour for activation and washed in DMEM medium. A suspension of 10^3^ CFU of killed S_Typhi treated previously with 37% formalin for 1-hour was applied during 3 hours at 37 °C, 5% CO_2_. Finally, the slides were washed, fixed in picric acid and stained using the Romanowsky method. Neutrophils were differentiated by “busy” aspect of nucleus with several lobes, while cells characterized by horseshoe-shaped nucleus with dishwater-gray cytoplasm and a few tiny granules were characterized as monocyte-macrophage lines. The percentage of phagocytic activity in cells was compared by multiple comparison by Tukey's method.


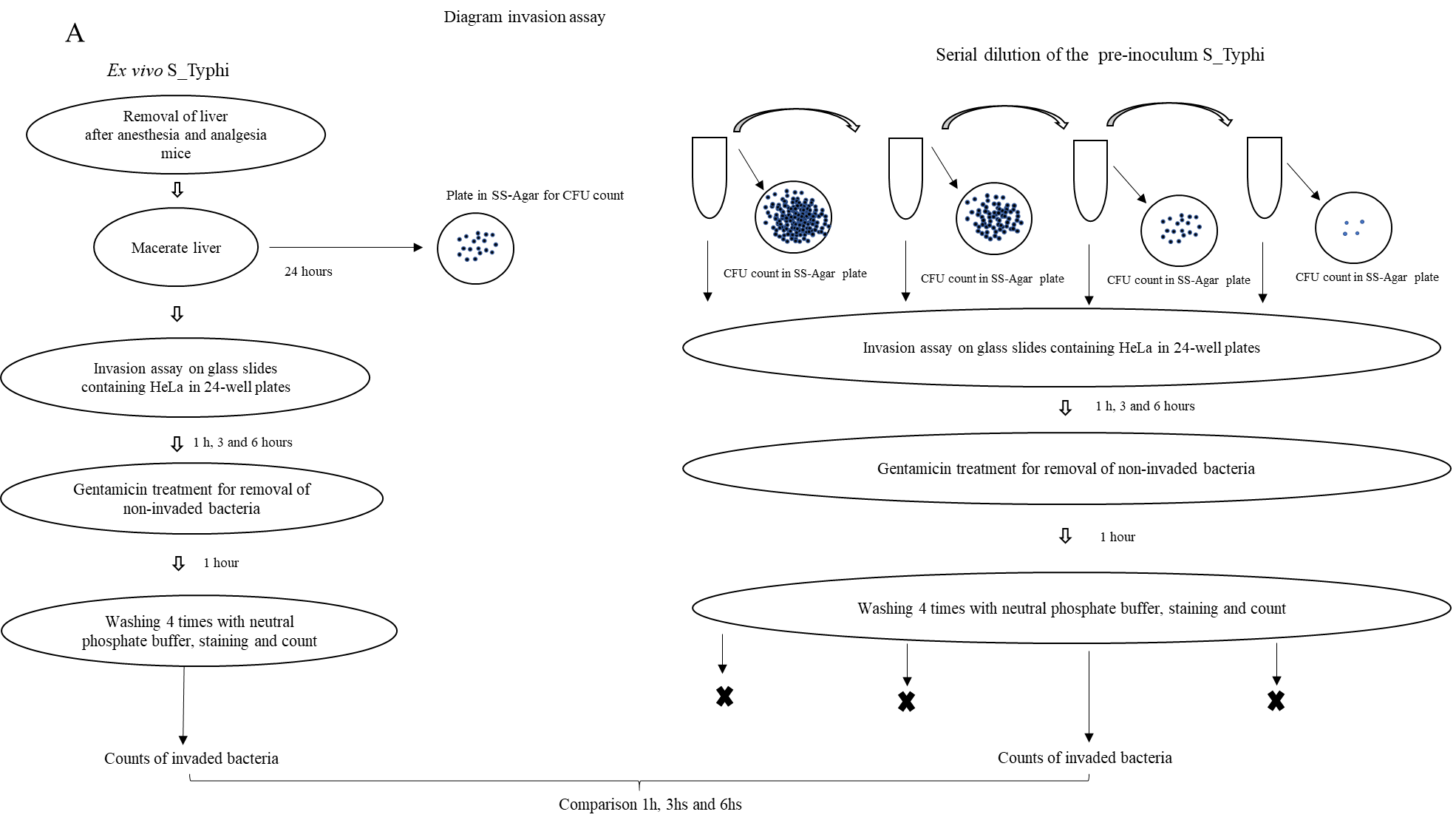
**Figure S4: The line diagram for clarifying the rationale of bacterial modulation.**

**Figure S4: The line diagram for clarifying the rationale of bacterial modulation.** Evaluation of the possibility of ex vivo Salmonella strains having been modulated after infection. A) Livers of mice that were coinfected on D4 were macerated and frozen at –80 ºC. Two ex vivo bacteria were thawed and set for the invasion assay in HeLa cells. The invasion time was one, three and six hours. After these periods, medium supplemented with gentamicin was added to the culture medium for one hour in order to eliminate external bacteria. Slides were then washed, stained using Romanowsky staining and bacteria were visualized. B) For comparison, an aliquot of S_Typhi was used under the same conditions as the pre-inoculum (in growth phase of nine hours in LB broth) and stored at –80 ºC for 15 days until the moment of the assay. HeLa cells were grown in 24-well plates containing glass slides (Knittel, Brazil) in DMEM medium with 10% fetal bovine (Sigma, Brazil) at 5% CO2 at 37 ºC until total confluence. Pre-inoculum bacteria were diluted in a series of 10-10 and sown in SS-Agar medium to define the inoculum equivalence of ex vivo bacteria. **X**= discarded data.

**5) Methods**

The analysis of epithelial integrity of the intestine and morphometric measurements were made using stereology of the Cavalieri principle of volume and the Delesse principle, both operated using the Imod program ^37^.

**Histopathological evaluation of the intestine:**  After euthanasia, the intestines of the animals were removed and kept in buffered formalin for 48 hours at room temperature. The samples were processed in the Quantitative Morphology Laboratory (LaMiq/UFAM). For this, the intestines were dehydrated in increasing concentrations of 70 and 96% ethanol (2 hours in each concentration), pre-infiltrated in 96% ethanol + hydroxyethyl methacrylate plastic historesin solution (Technovit 7100, Külzer-Heraues, Germany) overnight and infiltrated in 100% resin. The samples were arranged in individual Histobloc teflon molds (Külzer-Heraues, Germany) set in plastic resin + polymerizing solution. The molds were heated in an oven at 37 ºC until complete polymerization. The analysis of epithelial integrity of the intestine and morphometric measurements were made using stereology of the Cavalieri principle of volume and the Delesse principle, both operated using the Imod program ^37^.

**Determination of volume using the Cavalieri method**

Each block containing an intestine was observed in a stereomicroscope (Leica EZ4D Digital System, Germany). Its total length (L) was determined and this value was divided by six, representing the distance between sections that was marked on the block in order to be visualized during the microtomy (Supplementary Figure A). The microtomy was performed on a microtome (Leica RM 2145, Germany) and the sections subsequently stained with toluidine blue 0.5% (toluidine blue, 0.12 g; Na + borate, 0.5 g; distilled H_2_O, 100 mL) and basic fuchsin (basic fuchsin, 0.5 g and distilled H_2_O, 100 mL). The sections were arranged on histological slides (Supplementary Figure B) and photographed in low magnification (10) using a stereomicroscope (Leica EZ4D Digital System, Germany). The obtained images were analyzed in the program Imod version 4.7/stereology module (Kremer et al. 1996), where counting systems containing points were superimposed on the images (Figure below). Each time the points coincided with the wall of the intestine they were counted. The total volume of the intestine was obtained according to the Cavalieri principle ^38^. This principle is based on robust mathematical analysis, and its application is simple and extremely efficient. The volume of the intestine was calculated as follows: $\boldsymbol{V}\boldsymbol{(}\boldsymbol{mm}^{\boldsymbol{3}}\boldsymbol{)=}\sum_{\boldsymbol{i}\boldsymbol{=}\boldsymbol{1}}^{\boldsymbol{m}} \boldsymbol{Pi}\boldsymbol{\times}\boldsymbol{T}\boldsymbol{\times}\boldsymbol{a}\boldsymbol{/}\boldsymbol{p}$. (Gundersen et al. 1988). Where, ***V*** is the absolute volume of the intestine, $\sum_{\boldsymbol{i}\boldsymbol{=}\boldsymbol{1}}^{\boldsymbol{m}} \boldsymbol{Pi}$ is the total number of points in each section, ***a/p*** is the area represented by each point (41.607,84 μm^2^), and ***T*** (5.000 μm) is the distance between each section.

**Volume density using the Delesse principle**

The percentage of each component on the intestinal wall (mucous cells, mucous, submucosal, muscular and serous layer) was obtained using the Delesse principle (volume density) (Howard & Reed 2005). For this, an increase of 200 (Leica DM 500 microscope, Switzerland) was employed in randomly selected fields of view (Supplementary Figures D-E). The images obtained were analyzed using the program IMOD version 4.7/stereology module ^37^. The components were quantified by counting points and the result expressed in percentage as: $\boldsymbol{Vv}\boldsymbol{=}\frac{\sum_{\boldsymbol{i}\boldsymbol{=}\boldsymbol{1}}^{\boldsymbol{m}} \boldsymbol{Pcomp}}{\sum_{\boldsymbol{i}\boldsymbol{=}\boldsymbol{1}}^{\boldsymbol{m}} \boldsymbol{Pref}}$ where, ***Vv*** is the volume density (fractional volume or relative density) of a particular component of the intestine; *P comp* is the sum of points that touch the region of interest (mucosal cells, mucosal layer, submucosal, muscular and serous layer) and *P ref* is the sum of points that touch the reference space (all the intestinal wall). The percentage data (Vv) were transformed into absolutes when multiplied by the Cavalieri volume of the intestine: $\boldsymbol{Vabsoluto}\boldsymbol{=}\boldsymbol{VCavalieri}\boldsymbol{\times}\boldsymbol{Vv}$ ^39^.

**Surface density**

The surface area (S) of the mucous layer was determined on the same images used to determine the volume density ^38^. The images were superimposed with a test system containing pairs of lines for the estimation of surface density using the Buffon principle. Each time the lines intercept the mucous surface and the points touch the mucous lining epithelium, both are counted. The data from the intersection were used to determine the relationship of surface area-to-volume (*Sv*) as follows: $\boldsymbol{Sv} \left( \boldsymbol{mm}^{\boldsymbol{-}\boldsymbol{1}} \right)\boldsymbol{=}\frac{\boldsymbol{2}\sum_{\boldsymbol{i}\boldsymbol{=}\boldsymbol{1}}^{\boldsymbol{m}} \boldsymbol{I}}{\sum_{\boldsymbol{i}\boldsymbol{=}\boldsymbol{1}}^{\boldsymbol{m}} \boldsymbol{Pi}\boldsymbol{\times}\frac{\boldsymbol{l}}{\boldsymbol{p}}}$ Where: $\boldsymbol{2}\sum_{\boldsymbol{i}\boldsymbol{=}\boldsymbol{1}}^{\boldsymbol{m}} \boldsymbol{I}$ is the sum of the intersections of the lines and the test surfaces, $\sum_{\boldsymbol{i}\boldsymbol{=}\boldsymbol{1}}^{\boldsymbol{m}} \boldsymbol{,}\boldsymbol{Pi}$ is the sum of the points of touching the epithelium of the lining of the mucous membrane, and l/p is the length of the test line. The total surface area of each intestine was obtained by multiplying *Sv* by the Cavalieri volume of the intestine, according to the following equation: $\boldsymbol{S}\boldsymbol{urface} \boldsymbol{area} \left( \boldsymbol{mm}^{\boldsymbol{-}\boldsymbol{1}} \right)\boldsymbol{=}\boldsymbol{Sv} \boldsymbol{x} \boldsymbol{Cavalieri} \boldsymbol{Volume}$ ^39^.

# **Statistical analysis**

The estimate of the volume was determined according to Cavalieri’s principle, and the results were analyzed using one-way ANOVA (see Supplementary material .

$$\mathbf{CE}\mathbf{=} \left[ \boldsymbol{0}\boldsymbol{,}\boldsymbol{0724}\boldsymbol{\times}\frac{\boldsymbol{B}}{\sqrt{\boldsymbol{A}}}\boldsymbol{\times}\frac{\sqrt{\boldsymbol{n}}}{\left( \sum_{\boldsymbol{i}\boldsymbol{=}\boldsymbol{1}}^{\boldsymbol{m}} \boldsymbol{Pi} \right)^{\frac{\boldsymbol{3}}{\boldsymbol{2}}}} \right]^{\frac{\boldsymbol{1}}{\boldsymbol{2}}}$$

Where: **CE** indicates the error coefficient for determining the volume; $\frac{\boldsymbol{B}}{\sqrt{\boldsymbol{A}}}$, indicates the variance of the transverse areas (shape coefficient) and depends on the complexity of the forms of the structure; ***n*** represents the number of evaluated sections, and $\sum_{\boldsymbol{i}\boldsymbol{=}\boldsymbol{1}}^{\boldsymbol{m}} \boldsymbol{Pi}$ is the number of points counted on the sections.

The ***CE*** of volume and surface density was estimated according to using the equation:

$${\boldsymbol{CE}\boldsymbol{=}\left[ \frac{\boldsymbol{k}}{\boldsymbol{k}\boldsymbol{-}\boldsymbol{1}}\left\{ \frac{\sum\boldsymbol{u}^{\boldsymbol{2}}}{\sum\boldsymbol{u} \sum\boldsymbol{u}}\boldsymbol{+}\frac{\sum\boldsymbol{v}^{\boldsymbol{2}}}{\sum\boldsymbol{v} \sum\boldsymbol{v}}\boldsymbol{-}\boldsymbol{2}\frac{\sum\boldsymbol{uv}}{\sum\boldsymbol{u} \sum\boldsymbol{v}} \right\} \right]}^{\frac{\boldsymbol{1}}{\boldsymbol{2}}}$$

Where: ***CE*** indicates the error coefficient for determining the surface-to-volume or density-to-volume ratio; k represents the number of images analyzed and ***u*** and **v** are the totals of intersections or points made. A ***CE*** ≤ 10% is be considered satisfactory (accurate).

**6) Figure S5. Stereology of the volume of the small intestine and density of the volume of components in the intestinal wall**


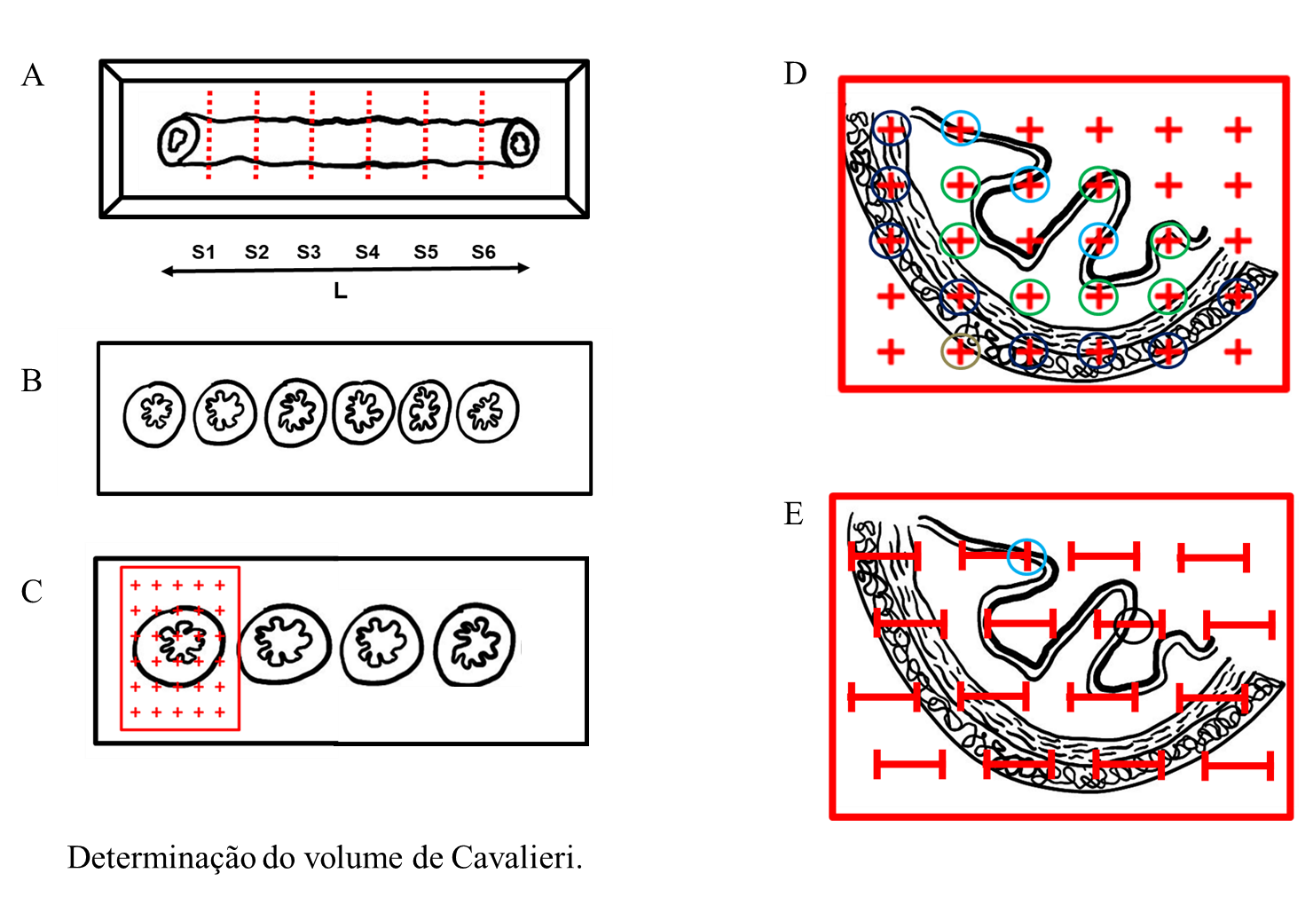


**Figure S5. Stereology of the volume of the small intestine and density of the volume of components in the intestinal wall.** (A-C) The total volume of the intestine was obtained according to the Cavalieri principle. (D-E) Count test systems containing dots and lines superimposed on diagrams of part of the intestine, measured by the percentage of the volume of mucous cells, mucous layer, submucosal, muscular and serous, which were obtained by the delesse principle. A) Intestine processed and set in plastic resin. Dashed lines indicate the distance between the serial sections (S). B) All sections (S) of an intestine were arranged on a single microscopic slide. C) Each section was photographed in its entirety and on it was superimposed a counting system containing points for the determination of the Cavalieri volume. D) Test system containing crosses for the estimation of volume density (Delesse). The central point of the cross is the reference for the count that will be computed whenever this overlaps the mucous layer (light blue circle), the submucosal layer (green circle), the muscular layers (dark blue circle) and the serous layer (gray circle). E) Test system containing pairs of lines for estimating surface density (Buffon). Counting is done in two stages: points touching the mucous layer and lines intercepting the edge of the mucous layer. In the scheme, a light blue circle indicates a point over the mucous layer, and the black circle indicates an intersection with the edge of the same layer.
